# Surgical Emergencies and Accidents

**Published:** 1884-11

**Authors:** 


### BOOK NOTICES.

SURGICAL EMERGENCIES AND ACCIDENTS. By J. G. Gilchrist, M. D., Professor of Surgical Pathology and Therapeutics in the State University of Iowa, etc., etc. Pp. 582; price, $4.50, cloth. Chicago: Duncan Brothers, 1884.

The practitioner who labors in the country or in small towns and villages is called upon to act as surgeon, physician, accoucheur, and, indeed, in almost every capacity! To such physicians will this work of Dr. Gilchrist be especially useful; and, indeed, we believe all of us will find it a convenient book to have at hand for ready reference. The object of the work is to treat of those surgical ailments which are not of sufficient gravity to require the aid of the specialist. Thus we find such subjects as these discussed: Contusions, wounds, effects of heat and cold, asphyxia, injuries to the blood vessels, nerves, muscles, eyes, ears, face, mouth, spine, throat, neck, chest, abdomen, genitals, etc. These are surgical cases with which the general practitioner very often has to deal; he will find them well treated of in this work. The present work is properly a companion volume to Dr. Gilchrist's *Surgical Therapeutics*, which is well known to most homœopathists.

